# Biographical

**Published:** 1891-08

**Authors:** 


### Biographical.

### WILLIAM H. ATKINSON, M.D., D.D.S.

The subject of this sketch was born January 23rd, 1815, at Newtown, Bucks County, Pennsylvania. His parents were of Quaker stock. His father, during the latter part of his life at least, was a minister in the Methodist Church. His mother, from a natural fitness and through some special preparation, engaged to some extent in medical practice, more particularly in her immediate neighborhood. Her success was said to have been unusually good. Their home, after some years, was changed from Bucks Co. to Mercer Co., Pennsylvania, which was then a new and sparsely settled part of the country.

The attainments and successful practice of his mother inclined his attention to the science and practice of medicine, though in his boyhood he was apprenticed to a tailor; this, and working upon the farm constituted his labor of early life. During this time he was studying and acquiring a good knowledge of nature in its varions phases and aspects, and doubtless here was laid the foundation of that thorough and extensive knowledge which he possessed, and ever after remained with him. In early life he showed unusual traits in the study and investigation of basal principles. About the time he came to full manhood he went to Meadville, Penn., and there began a thorough and systematic course of the study of medicine with Dr. Wm. Woodruff. After attaining a good degree of medical knowledge he practiced in connection with his preceptor. Things went on thus for a time, after which he sought a more intimate connection with the family of his preceptor, which resulted in his marriage to Martha C. Woodruff, May 17th, 1840.

He graduated in medicine at Willoughby University, Willoughby, Ohio, in 1847, at the age of thirty-two. For that occasion he wrote a thesis on the subject of "sleep," a paper which attracted considerable attention at the time. He practiced at Meadville in partnership with his father-in-law for some time after his graduation and gave special attention to surgery and attained considerable reputation in this line of practice. During the time of this practice he made the acquaintance of an itinerant dentist, and became familiar with the practice of this department. It especially attracted his attention, and he thus early, realized the fact that dental practice ought to be much more than a mere trade. His appreciation of it grew as time passed on and his interest in it increased until it largely absorbed his thoughts. He by degrees, and almost imperceptibly, entered the practice of dentistry, and he manifested an effort to make it an ennobling profession; relinquishing the practice of medicine gradually as he took up the practice of dentistry.

About this time he removed to Norwalk, Huron county, Ohio. He there practiced successfully as a surgeon, physician and dentist. He remained in Norwalk two or three years. In 1853 he removed to Cleveland, Ohio, where he formed a partnership with Dr. F. S. Slosson, then a leading practitioner of dentistry in Cleveland. He here manifested such energy and aggressive industry, in his new occupation as to soon become known as one of the most thorough and progressive members of the profession.

While in practice with Dr. Slosson he received, as a student, Dr. Charles R. Butler—his first student. After thoroughly training him as a pupil he formed a partnership with him in practice. Dr. Atkinson received the degree of Doctor of Dental Surgery in the Ohio College of Dental Surgery in Cincinnati in 1859. He became greatly interested in the growth and progress of his newly chosen profession, and it may be said that it was in a condition to greatly need just such support as he was able, and did give it. He was enthusiastic in the formation of dental and scientific societies, throwing his whole energy, whenever opportunity offered, into such enterprises. He was also greatly interested in the subject of dental education, ever ready to give wise counsel and aid, whenever it was in his power and wherever needed.

It is said, and we think with truth, that he inaugurated operative clinics before dental societies; his first effort in this direction being before the Indiana State Dental Society in 1859. He there introduced and first drew the attention of the dental profession to the use of the mallet in filling teeth. By his enthusiasm he awakened interest and stimulated thought wherever he went. He was a student in all natural sciences; he was greatly interested in microscopy, and was instrumental in extending its use in various lines of investigation in dental science.

His professional life was a gratifying success; success in its highest aspect.

In 1861 he removed to the City of New York, a larger field there being opened for the exercise for his great ability. For one year after his removal to that city he had charge of the large dental depot of the S. S. White Co. That being less congenial to his tastes he returned to the practice of his profession at the end of the time designated, where he established a most enviable position, not only as a practitioner of dentistry, but as one who for years exercised an almost unparalleled influence in the profession, and this too in the way of aiding and making better professionally those who came within the sphere of his influence; this he did, not only at his home and at his office, but he visited all parts of the country laboring to organize, establish and build up his chosen profession.

He was the first to promulgate many new points in practice; he never hesitated to put forth anything that he thought would be of service to others. He communicated freely all he had; he did more, perhaps, to elevate the profession by introducing a higher appreciation for thorough work than anyone else in this country; for example, when he removed to Cleveland he found there in the hands of even the best, that professional fees were at the tinker's standard, and the quality of the operations corresponded. He promptly more than doubled the average fees that were charged and made his operations to correspond. He received sharp criticism, even rebuke for this, but it did not turn him from his course, but it ultimately served the purpose of bringing others up to a far better and higher standard of both work and fees.

He possessed a great faculty for communicating knowledge to others, as was shown in the fact, that for years he had private classes that came to his office at set times, and sat under his instruction, and such was his power in this work that he was able to communicate not only largely of his own knowledge and ability, but of his enthusiasm as well, to all those who were his pupils, as many could bear witness to this day.

Dr. Atkinson gave much effort and influence for the founding and organization of dental societies in various parts of the country. He was one of the founders of the American Dental Association of which he continued an active member till his death; he was its first president after its organization. He was also largely instrumental in the organization of the First District Dental

Society of New York, and also of the Brooklyn Dental Society. He was an active member in many dental societies in the country and was an honorary member in every other one of any considerable importance.

In all his association work everywhere he was the means of enkindling an enthusiasm that was unequalled by that of any other member of the profession. His work, his speaking, and his writing always induced men to earnest thought.

He was also instrumental in the organization of the New York College of Dentistry, in which for a time he was a teacher. Everything he did and every resource he possessed was made subservient to his ambition for the advancement of dental science and art. Some of the best men in the dental profession owe much to Dr. Atkinson for what they are to-day. He ever had a watchful care and interest in all that pertained to the progress and up-building of his chosen profession; always jealous for its interests and strongly antagonistic to everything derogatory to its welfare.

Within three years before his death he suffered grievous affliction; his wife, his eldest and youngest sons and three grandchildren passed from this life. These great afflictions seemed almost to crush him, and no doubt hastened the termination of his earthly career. He was a person of great sympathy and of an exceedingly sensitive nature, though of a strong will, which enabled him oftentimes to hide feeling that was raging like a storm within.

He will long be missed in the profession; hundreds of eyes will be turned to his vacant seat in the meetings and convocations where he has been in the past almost a constant attendant and participator.

Let all profit by the noble example which he in so many ways set before his fellow men.

